# Unique chemosensitivity of MAC 16 tumours to flavone acetic acid (LM975, NSC 347512).

**DOI:** 10.1038/bjc.1988.215

**Published:** 1988-09

**Authors:** M. C. Bibby, J. A. Double, P. M. Loadman

**Affiliations:** Clinical Oncology Unit, University of Bradford, U.K.

## Abstract

**Images:**


*Br. J. Cancer* (1988), **58**, 341–344 © The Macmillan Press Ltd., 1988

### Unique chemosensitivity of MAC 16 tumours to flavone acetic acid (LM975, NSC 347512)

M.C. Bibby, J.A. Double & P.M. Loadman

*Clinical Oncology Unit, University of Bradford, Bradford BD7 1DP, UK.*

**Summary** MAC 16 is one of a series of mouse colon tumours originally induced by dimethylhydrazine. It is a relatively slow growing subcutaneous adenocarcinoma which becomes necrotic as it grows and causes severe body wasting in the host. This study has indicated that the tumour is resistant to a large number of standard anti-cancer drugs but is highly responsive to the investigational agent flavone acetic acid (FAA). The levels of FAA achieved in tumours are lower than those necessary for activity *in vitro* suggesting its mechanism of action *in vivo* is not direct cytotoxicity. Responding tumours demonstrate massive tissue necrosis and those which are not cured have viable tumour cells associated with tumour blood vessels. The anti-tumour effects are accompanied by control of the host's cancer cachexia. The unique chemosensitivity of MAC 16 to FAA suggests that this agent has a novel mechanism which may be dependent upon specific biological characteristics of tumours.

Flavone acetic acid (FAA) is a novel anti-tumour agent under early clinical investigation in Europe and the USA. The rationale for the submission of this compound for clinical trial was significant anti-tumour effects in solid murine tumours (Corbett *et al.*, 1986; Plowman *et al.*, 1986; Bibby *et al.*, 1987*a*). Initial clinical investigations have demonstrated that FAA is also devoid of bone marrow toxicity (Kerr *et al.*, 1986). MAC 16 is one of a panel of mouse adenocarcinomas of the colon initially induced by 1,2-dimethylhydrazine (Haase *et al.*, 1973). The tumour system has been extensively used in experimental chemotherapy studies but the chemosensitivity of MAC 16 has not been fully evaluated. Preliminary chemosensitivity studies with MAC 16 (Ali *et al.*, 1985) demonstrated that the tumour was moderately responsive to 5-fluorouracil (5FU) and cyclophosphamide but did not respond to methyl-CCNU (MeCCNU) or mitozolomide. Implantation of MAC 16 results in a significant loss of host body weight without excessive tumour mass and without a concomitant reduction of food intake by the host (Bibby *et al.*, 1987*b*). The present study examines the anti-tumour effects of FAA and a series of standard anti-cancer agents on MAC 16 and also investigates plasma and tumour levels of FAA.

### Materials and methods

### *Animals*

Pure strain NMRI mice (age 6–8 weeks) from our inbred colony were used. They were fed on CRM Diet (Labsure, UK) and water *ad libitum*.

### *Test compounds*

FAA was a gift from Lipha (Lyon, France) via Prof. S.B. Kaye, University of Glasgow, UK; MeCCNU and DTIC were gifts from the National Cancer Institute, USA. Mitozolomide was a gift from Prof. M.F.G. Stevens, University of Aston, UK; ThioTEPA from Lederle Laboratories, Gosport, Hants, UK; 5-FU from Roche, Welwyn Garden City, UK; cyclophosphamide from the Boehringer Corpor ation, London, UK and chlorambucil from Dr D.E.V. Wilman, Institute of Cancer Research, Sutton, UK. FAA, cyclophosphamide, thioTEPA and 5-FU were dissolved in 0.9% sterile saline. Chlorambucil and MeCCNU were dissolved in 10% ethanol/arachis oil, and DTIC and mitozolomide were suspended in arachis oil. Drugs were prepared at appropriate concentrations for a desired dose to be administered in 0.1 ml per 10 g body weight. Drugs were made up immediately before use and all injections were i.p.

### *Tumours*

The development of several transplantable adenocarcinomas of the large bowel in mice from primary tumours induced by prolonged administration of 1,2,dimethylhydrazine has been described elsewhere (Double *et al.*, 1975). MAC 16 tumours were excised from donor animals and placed in sterile 0.9% saline containing streptomycin ($2\,mg\,ml^{-1}$) and penicillin ($2000\,U\,ml^{-1}$) and cut into small fragments $\sim 1 \times 2$ mm in size. Fragments were implanted s.c. into the flank by means of a trocar. Take rates are variable with good rates being dependent on careful implantation of viable tumour fragments. Positive takes can only be identified 2–3 weeks after implantation.

### *Chemotherapy*

Tumour bearing animals were allocated by restricted randomisation into groups of 10. Chemotherapy did not commence until the tumours could be reliably measured, i.e. until they achieved minimum dimensions of $4 \times 5$ mm. Therapeutic effects were assessed by twice weekly 2-dimensional caliper measurements of the tumour. Tumour volume was calculated from the formula $a^2 \times b/2$ where a is the smaller diameter and b is the larger (Geran *et al.*, 1972). Tumour volumes were normalised with respect to starting volumes and graphs of the relative tumour volumes against time were plotted on semi-log graph paper.

### *Measurement of drug levels in plasma and MAC 16 tumours*

*Reagents* Spectroscopic grade ethanol (BDH Chemicals, Poole, Dorset, UK) p-dimethylaminobenzaldehyde (Sigma Chemical Co., Poole, Dorset, UK) and triple distilled water were used. Other agents were of analytical grade.

*Sample collection* Blood samples from three tumour bearing mice at each time point were taken by cardiac puncture under ether anaesthesia, collected into heparinised tubes, centrifuged at 2000*g* and 4°C for 10 min and then separated plasma was stored at −20° until analysis. The mice were killed by cervical dislocation and rapidly dissected. Tumours were immediately frozen in liquid nitrogen and stored at −20°C.

*Sample extraction and chromatography* FAA was extracted from fluid samples using solid phase chromatography and measured by an HPLC method described by Double *et al.* (1986) and modified from Kerr *et al.* (1985). Tumour

Correspondence: M.C. Bibby.
Received 21 January 1988; and in revised form, 13 May 1988.

samples were mixed with 0.1 M sodium acetate–acetic acid buffer, pH4 (10% w/v) and homogenised using an Ultra-turrax blender. Homogenates were centrifuged at 2500$g$ and 4°C for 5 min. The supernatants were separated and after the addition of internal standard, p-dimethylaminobenzaldehyde (100 $\mu$l at 100 $\mu$g ml$^{-1}$) were extracted for analysis. Standard curves were prepared by the addition of FAA to buffered control mouse plasma (pH4) and plotting ratio of peak areas of FAA to the internal standard against drug concentration. Peaks were traced and integrated with an Isaac Model 42A data module (Syborg Corporation, USA), an Apple IIE Computer (Apple Computer Inc., USA) and Appligration II Software (Dynamic Solutions Corporation, USA). The curves were linear over the range 0.4 to 40 $\mu$g ml$^{-1}$. The assay was sensitive to drug concentrations down to 10 ng ml$^{-1}$ and recovery was >90%.

*Pharmacokinetic analysis* The area under the concentration versus time curve (AUC) was calculated for plasma and tumour samples using the trapezoid rule.

### Results

MAC 16 is a moderately well differentiated adenocarcinoma of the colon which has been serially passaged for a period of 8 years. During this time the tumour has remained histologically unaltered. A small degree of central necrosis is present in small tumours and this increases with tumour size. The effects of a series of anti-cancer agents against MAC 16 at or close to maximum tolerated dose are presented in Table I. No significant growth delay was demonstrated with any of these agents at any dose level. Tolerance to chlorambucil, DTIC and MeCCNU and ThioTEPA was lower in female mice bearing MAC 16 tumours than in normal female NMRI mice.

The effects of FAA against MAC 16 are shown in Figure 1. A dose of 300 mg kg$^{-1}$ is acutely toxic with 4 out of 10 deaths occurring in this group. Treatment with 200 mg kg$^{-1}$ on 0 and day 7 results in a highly significant growth delay with 8 out of 10 mice in this group being cured and no drug associated deaths. ThioTEPA at the $LD_{10}$ produces a very short cessation of tumour growth but tumour growth rapidly returns to control level.

Growth of MAC 16 in untreated animals is accompanied by a dramatic body weight loss (Figure 2). Treatment with FAA causes a rapid recovery in body weight. The interim anti-tumour effect caused by ThioTEPA at the $LD_{10}$ is also accompanied by a cessation of body weight loss, but the rapid regrowth of the tumour is accompanied by a drop in body weight until this group reaches the same mean body weight as the untreated control group.

The histology of MAC 16 tumours which respond to FAA but are not cured is shown in Figure 3. The normal histology of the appropriate passage is shown in Figure 3a and the effects of FAA on tumours of that passage are shown in Figures 3b and 3c. The viable tumour cells are associated with vessels within the tumour whereas the vast

**Table I** Effects of a series of standard anti-cancer agents against MAC 16

| *Drug* | *Dose (mg kg$^{-1}$)* | *Drug associated deaths* | *MTD in normal female mice (mg kg$^{-1}$)* | *Tumour growth delay (days)* |
|---|---|---|---|---|
| Chlorambucil | 20 | 1/10 | 30 | 0 |
| Cyclophosphamide | 300 | 2/10 | 300 | 0 |
| DTIC | 200 | 2/10 | 300 | 0 |
| 5FU | 120 | 2/10 | 120 | 0 |
| MeCCNU | 25 | 0/10 | 30 | 0 |
| Mitozolomide | 37.5 | 0/10 | 40 | 0 |
| ThioTEPA | 15 | 1/10 | 20 | 0 |

**Figure 1** Anti-tumour effects of flavone acetic acid and thio-TEPA against established MAC 16 tumours.

**Figure 2** Effects of flavone acetic acid and thioTEPA on mean body weight of MAC 16 bearing mice.

majority of the tumour cells are dead. Levels of FAA in the plasma and MAC 16 tumours following i.p. administration of 200 mg kg$^{-1}$ are given in Figure 4. AUCs are 2.17 mg h ml$^{-1}$ and 0.50 mg h gl$^{-1}$ for plasma and tumour tissue respectively.

### Discussion

Chemotherapy studies have demonstrated that MAC 16 is unresponsive to a series of standard anti-cancer drugs. Previous earlier chemosensitivity studies demonstrated that early passages of MAC 16 responded modestly to 5FU and cyclophosphamide (Ali *et al.* 1985) but mice bearing these tumours were able to tolerate similar levels of these agents as normal mice. The serial passage of the MAC 16 tumour line has been biased by selection for the cachexia caused in tumour bearing mice (Bibby *et al.* 1987*b*) and this appears to

**Figure 3** Histological effects of treatment of MAC tumours with flavone acetic acid: **a** untreated, **b** regrowing tumour after a long growth delay, **c** same tumour showing viable tumour cells associated with a tumour blood vessel.

**Figure 4** Levels of FAA in plasma (●) and MAC 16 tumours (▲) following a single i.p. injection of 200 mg kg$^{-1}$ (plasma, $\mu$g ml$^{-1}$; tumour, $\mu$g g$^{-1}$).

have influenced responses and drug tolerance. There are no major histological differences between early and late tumour passages and the degree of necrosis of the tumour relates to tumour size. The histology, growth characteristics and resistance of this tumour suggests it may be a clinically relevant model for the evaluation of potential anti-cancer drugs.

The i.p. maximum tolerated dose (MTD) of FAA in female NMRI mice bearing MAC 16 tumours was 200 mg kg$^{-1}$ whereas the MTD in non-tumour bearing mice and those bearing other tumours of the MAC system was 300 mg kg$^{-1}$ (Bibby *et al.* 1987*a*). Previous experimental chemotherapy studies have shown that FAA is most active against other subcutaneous MAC tumours when administered by a two dose schedule split by seven days (Bibby *et al.* 1987*a*). FAA is highly active against MAC 16 tumours by this treatment schedule. Control of tumour growth is accompanied by a control of the tumour associated cachexia. The small anti-tumour effect demonstrated by thioTEPA at a toxic dose is also accompanied by a small effect on host body weight. The unique chemosensitivity of MAC 16 to FAA demonstrated in this study strengthens the suggestion of Corbett *et al.* (1986) that for the first time an agent has been identified with a very broad, perhaps nearly universal solid tumour activity.

The pharmacokinetic data in this study following a dose of 200 mg kg$^{-1}$ FAA suggests that the anti-tumour effects are not the result of a direct cytotoxic mechanism. Previous *in vitro* studies with a variety of tumour cell lines indicate that high drug concentrations and long exposure times are necessary to achieve direct cytotoxicity (Bibby *et al.*, 1987*a*; Capolonga *et al.*, 1987; Schroyens *et al.*, 1987). Preliminary studies in this laboratory have also demonstrated that MAC 16 cells are resistant to FAA *in vitro* at experimentally achievable drug levels *in vivo*.

Histological examination of MAC 16 tumours which are not cured but are beginning to regrow after a long growth delay demonstrates massive tumour necrosis with varying degrees of haemorrhage. Viable cells are seen in close proximity to tumour blood vessels.

Several possible mechanisms have now been described for the action of FAA in subcutaneous tumours. Smith *et al.* (1987) have described the induction of haemorrhagic necrosis in mouse colon 26 and colon 38 tumours and suggest that FAA may work in a similar fashion to tumour necrosis factor (TNF). Ching and Baguley (1987) and Wiltrout (1987) have also suggested that natural killer (NK) cells may be involved in the mechanisms of action of FAA as it activates NK cell activity in mice.

In preliminary studies in this laboratory with another tumour line (Bibby *et al*,. 1987*c*) we have demonstrated that tumour vasculature may be important in the action of FAA as the anti-tumour effects become more marked as vascular composition of viable tumour increases with time after implantation. This may be simply because of better drug delivery to established tumours but may also be an indication that tumour vasculature itself is involved as a component of therapy. Studies on the role of tumour blood vessels in the response to FAA are currently being undertaken. Rubin *et al.* (1987) have suggested that anti-tumour effects in mice may be related to altered platelet function following FAA treatment.

In conclusion FAA is highly active in a mouse colon tumour which is resistant to a number of standard anti-cancer drugs. The tolerance of MAC 16 tumour bearers to standard agents and FAA is impaired but responses are still achieved with FAA. The anti-tumour effects are accompanied by control of the tumour associated cachexia. The low tumour concentrations and the unique chemosensitivity suggest that FAA is working by a completely novel mechanism for a chemotherapeutic agent. The plasma profiles associated with activity against subcutaneous tumours in mice have now been attained in man (Kerr *et al.*, 1987) but no responses have yet been seen suggesting that low dose activity may be dependant upon specific biological characteristics of experimental subcutaneous tumours.

Supported by Whyte Watson/Turner Cancer Research Trust, Bradford.

### References

ALI, S.A., BIBBY, M.C. & DOUBLE, J.A. (1985). Body weight loss (cancer cachexia) following transplantation of an adenocarcinoma of the mouse colon (MAC 16). *Br. J. Cancer*, **52,** 452.

BIBBY, M.C., DOUBLE, J.A., PHILLIPS, R.M. & LOADMAN, P.M. (1987*a*). Factors involved in the anti-cancer activity of the investigational agents LM985 (flavone acetic acid ester) and LM975 (flavone acetic acid). *Br. J. Cancer*, **55,** 159.

BIBBY, M.C., DOUBLE, J.A., ALI, S.A., FEARON, K.H., BRENNAN, R.A. & TISDALE, M.J. (1987*b*). Characterisation of a transplantable adenocarcinoma of the mouse colon producing cachexia in recipient animals. *J. Natl Cancer Inst.*, **78,** 539.

BIBBY, M.C., DOUBLE, J.A., PHILLIPS, R.M., LOADMAN, P.M. & GUMMER, J.A. (1987*c*). Experimental anti-tumour effects of flavone acetic acid (LM975). In *Plant Flavonoids in Biology and Medicine II.* Biochemical Cellular and Medicinal Properties. Alan R. Liss: New York. In press.

CAPOLONGA, L.S., BALCONI, G., UBEZIO, P. & 5 others (1987). Antiproliferative properties of flavone acetic acid (NCS 347512) (LM975) a new anticancer agent. *Eur. J. Cancer Clin. Oncol.*, **23,** 1529.

CHING, L.M. & BAGULEY, B.C. (1987). Introduction of natural killer cell activity by the antitumour compounds flavone acetic acid (NSC 347512). *Eur. J. Cancer Clin. Oncol.*, **23,** 1047.

CORBETT, T.H., BISSERY, M.C., WOZNIAK, A. & 5 others (1986). Activity of flavone acetic acid (NSC 347512) against solid tumours of mice. *Investigational New Drugs*, **4,** 207.

DOUBLE, J.A., BALL, C.R. & COWEN, P.M. (1975). Transplantation of adenocarcinoma of the colon in mice. *J. Natl Cancer Inst.*, **54,** 271.

DOUBLE, J.A., BIBBY, M.C. & LOADMAN, P.M. (1986). Pharmacokinetics and anti-tumour activity of LM985 in mice bearing transplantable adenocarcinomas of the colon. *Br. J. Cancer*, **54,** 595.

GERAN, R.I., GREENBERG, N.H., MACDONALD, M.M., SCHUMACHER, M. & ABBOT, B.J. (1972). Protocol for screening chemical agents and natural products against tumours and other biological systems (third edition). *Cancer Chemother. Rep.*, **3,** 1.

HAASE, P., COWEN, D.M., KNOWLES, J.C. & COOPER, E.H. (1973). Evaluation of dimethylhydrazine-induced tumours in mice as a model system for colorectal cancer. *Br. J. Cancer*, **24,** 530.

KERR, D.J., KAYE, S.B., CASSIDY, J. & 6 others (1985). A clinical pharmacokinetic study of LM985 and LM975. *Br. J. Cancer*, **52,** 467.

KERR, D.J., KAYE, S.B., CASSIDY, J. & 8 others (1987). Phase I and pharmacokinetic study of flavone acetic acid. *Cancer Res.*, **47,** 6776.

KERR, D.J., KAYE, S.B., GRAHAM, J. & 8 others (1986). Phase I and pharmacokinetic study of LM985 (flavone acetic acid ester). *Cancer Res.*, **46,** 3142.

PLOWMAN, J., NARAYANAN, V.L., DYKES, D. & 4 others (1986). Flavone acetic acid. A novel agent with preclinical antitumour activity against colon adenocarcinoma 38 in mice. *Cancer Treat Rep.*, **70,** 631.

RUBIN, J., AMES, M.M., SCHULL, A.J., NICHOLS, W.J., BOWIE, E.J.W. & KOVACH, J.S. (1987). Flavone acetic acid inhibits induced platelet agglutination and prolongs bleeding time. *Lancet*, **ii,** 1081.

SCHROYENS, W.A., DODION, P.P., SANDERS, C. & 5 others (1987). *In vitro* chemosensitivity testing of flavone acetic acid (LM975 NSC 347512) and its diethylaminoethyl ester derivative (LM985; NSC 293015). *Eur. J. Cancer Clin. Oncol.*, **23,** 1135.

SMITH, G.P., CALVERLEY, S.B., SMITH, M.J. & BAGULEY, B.C. (1987). Flavone acetic acid (NSC 347512) induces haemorrhagic necrosis of mouse colon 26 and 38 tumours. *Eur. J. Cancer Clin. Oncol.*, **23,** 1209.

WILTROUT, R.H. (1987). Systemic augmentation of natural killer (NK) activity by the chemotherapeutic drug flavone 8-acetic acid. *Proc. Am. Assoc. Cancer Res.*, **28,** 347 (*Abstract*).


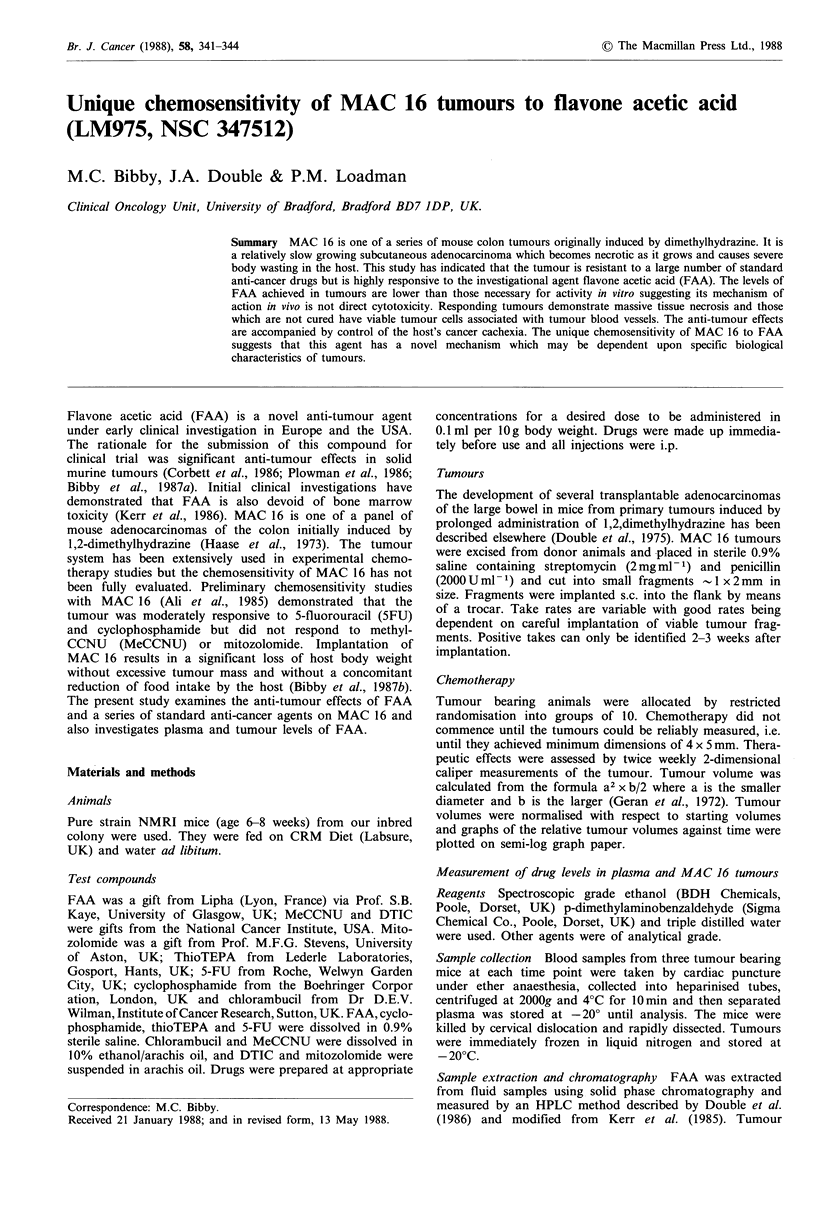

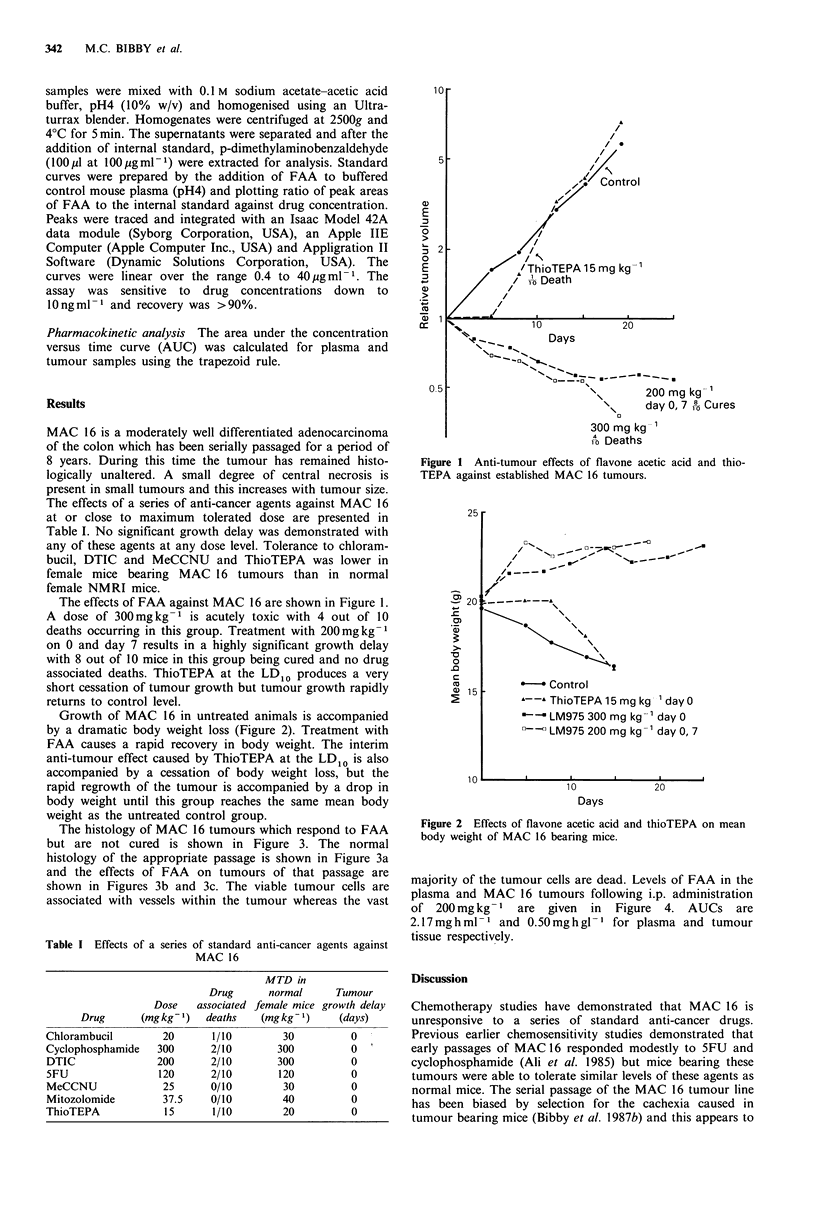

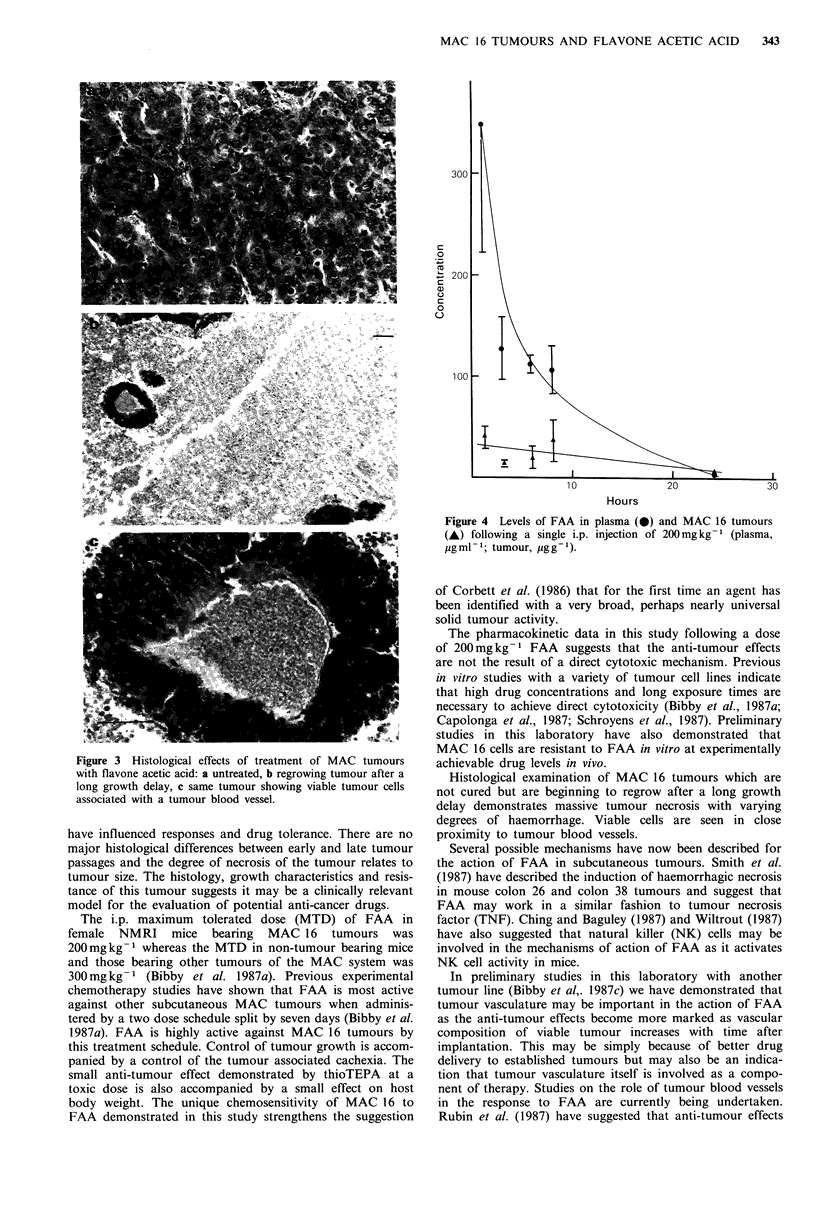

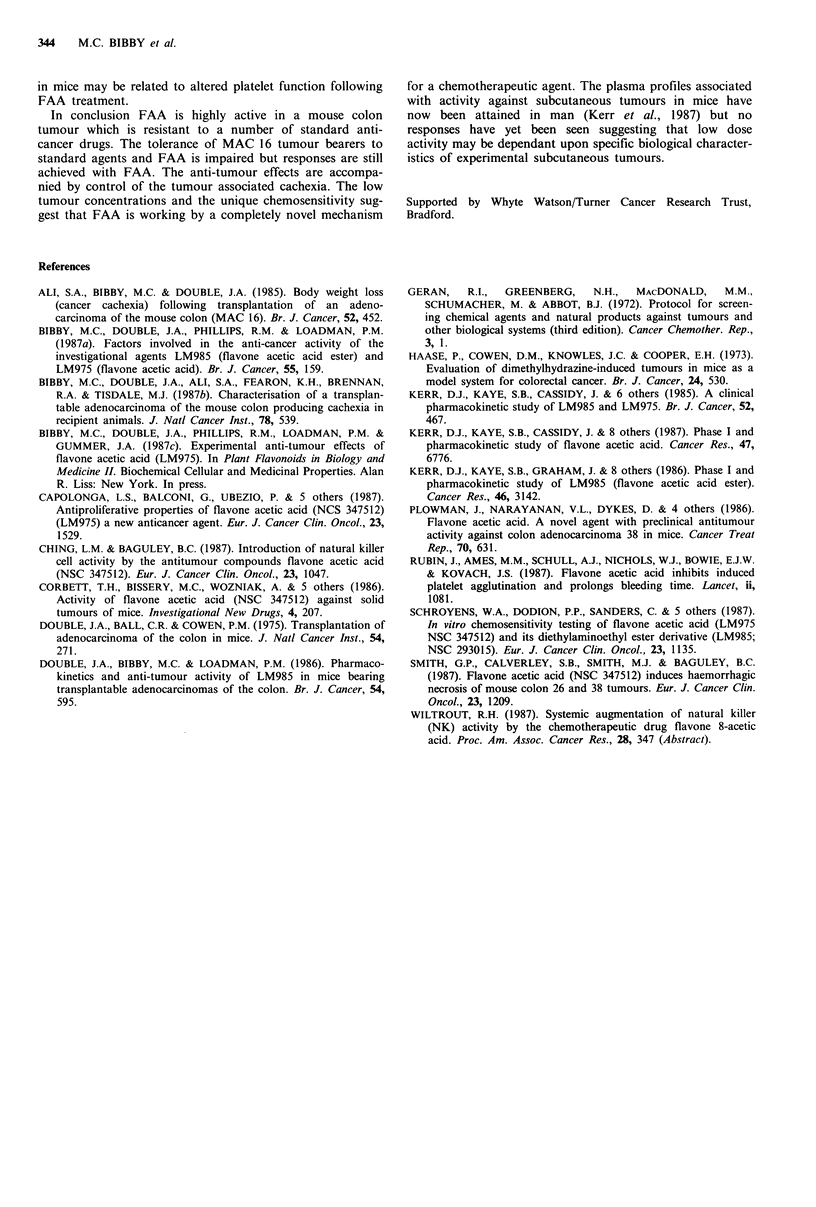


## References

[OCR_00420] Bibby M. C., Double J. A., Ali S. A., Fearon K. C., Brennan R. A., Tisdale M. J. (1987). Characterization of a transplantable adenocarcinoma of the mouse colon producing cachexia in recipient animals.. J Natl Cancer Inst.

[OCR_00414] Bibby M. C., Double J. A., Phillips R. M., Loadman P. M. (1987). Factors involved in the anti-cancer activity of the investigational agents LM985 (flavone acetic acid ester) and LM975 (flavone acetic acid).. Br J Cancer.

[OCR_00433] Capolongo L. S., Balconi G., Ubezio P., Giavazzi R., Taraboletti G., Regonesi A., Yoder O. C., D'Incalci M. (1987). Antiproliferative properties of flavone acetic acid (NSC 347512) (LM 975), a new anticancer agent.. Eur J Cancer Clin Oncol.

[OCR_00439] Ching L. M., Baguley B. C. (1987). Induction of natural killer cell activity by the antitumour compound flavone acetic acid (NSC 347 512).. Eur J Cancer Clin Oncol.

[OCR_00444] Corbett T. H., Bissery M. C., Wozniak A., Plowman J., Polin L., Tapazoglou E., Dieckman J., Valeriote F. (1986). Activity of flavone acetic acid (NSC-347512) against solid tumors of mice.. Invest New Drugs.

[OCR_00449] Double J. A., Ball C. R., Cowen P. N. (1975). Transplantation of adenocarcinomas of the colon in mice.. J Natl Cancer Inst.

[OCR_00454] Double J. A., Bibby M. C., Loadman P. M. (1986). Pharmacokinetics and anti-tumour activity of LM985 in mice bearing transplantable adenocarcinomas of the colon.. Br J Cancer.

[OCR_00467] Haase P., Cowen D. M., Knowles J. C., Cooper E. H. (1973). Evaluation of dimethylhydrazine induced tumours in mice as a model system for colorectal cancer.. Br J Cancer.

[OCR_00477] Kerr D. J., Kaye S. B., Cassidy J., Bradley C., Rankin E. M., Adams L., Setanoians A., Young T., Forrest G., Soukop M. (1987). Phase I and pharmacokinetic study of flavone acetic acid.. Cancer Res.

[OCR_00482] Kerr D. J., Kaye S. B., Graham J., Cassidy J., Harding M., Setanoians A., McGrath J. C., Vezin W. R., Cunningham D., Forrest G. (1986). Phase I and pharmacokinetic study of LM985 (flavone acetic acid ester).. Cancer Res.

[OCR_00487] Plowman J., Narayanan V. L., Dykes D., Szarvasi E., Briet P., Yoder O. C., Paull K. D. (1986). Flavone acetic acid: a novel agent with preclinical antitumor activity against colon adenocarcinoma 38 in mice.. Cancer Treat Rep.

[OCR_00493] Rubin J., Ames M. M., Schutt A. J., Nichols W. L., Bowie E. J., Kovach J. S. (1987). Flavone-8-acetic acid inhibits ristocetin-induced platelet agglutination and prolongs bleeding time.. Lancet.

[OCR_00499] Schroyens W. A., Dodion P. F., Sanders C., Loos M., Dethier N. E., Delforge A. R., Stryckmans P. A., Kenis Y. (1987). In vitro chemosensitivity testing of flavone acetic acid (LM975; NSC 347512) and its diethylaminoethyl ester derivative (LM985; NSC 293015).. Eur J Cancer Clin Oncol.

[OCR_00505] Smith G. P., Calveley S. B., Smith M. J., Baguley B. C. (1987). Flavone acetic acid (NSC 347512) induces haemorrhagic necrosis of mouse colon 26 and 38 tumours.. Eur J Cancer Clin Oncol.

